# Seroepidemiological Characteristics and Influencing Factors of Hepatitis B Virus Infection Among Pregnant Women in Nanning City, China (2024–2025): A Cross-Sectional Study

**DOI:** 10.3390/vaccines14090765

**Published:** 2026-09-01

**Authors:** Chang Huang, Tao Lan, Bin Xu, Qiuyun Deng, Chi Tang

**Affiliations:** 1 Nanning Center for Disease Control and Prevention, Nanning 530023, China; huangchang130@163.com (C.H.);; 2 Nanning Medical Key Discipline of Infectious Disease Prevention and Control, Nanning 530023, China; 3 Guangxi Zhuang Autonomous Region Center for Disease Control and Prevention, Nanning 530023, China; 4 Nanning Municipal Health Commission, Nanning 530023, China

**Keywords:** pregnant women, Hepatitis B virus, seroepidemiology, influencing factors, mother-to-child transmission prevention, China

## Abstract

**Objectives:** This cross-sectional study characterized HBV serological profiles and associated factors among pregnant women in Nanning to inform targeted MTCT prevention strategies. **Methods:** A total of 1935 pregnant women were enrolled via stratified cluster random sampling from October 2024 to May 2025. Five HBV serological markers were quantified by chemiluminescence immunoassay. Multivariable analysis identified factors associated with HBsAg, HBsAb, and HBcAb positivity. HBsAb geometric mean concentration (GMC) and ratio (GMR) were calculated. **Results:** The overall seropositivity rates (with 95% CI) of HBsAg, HBsAb, HBeAg, HBeAb, and HBcAb were 6.98% (5.92–8.20%), 59.48% (57.28–61.65%), 1.50% (1.05–2.14%), 15.50% (13.96–17.18%), and 33.02% (30.96–35.15%), respectively. HBsAg, HBeAb, and HBcAb positivity increased with age. The HBsAb GMC among uninfected pregnant women was 4.63 mIU/mL (95% CI: 3.72–5.75). Rural pregnant women had lower HBsAb seropositivity than urban counterparts. Unvaccinated women or those with unknown vaccination status showed higher HBsAg, HBeAg, HBeAb, and HBcAb positivity, and lower HBsAb seropositivity, compared with vaccinated women. Advanced age, chronic comorbidities, and HBV exposure were risk factors for HBsAg positivity, whereas HBV vaccination was protective. For HBsAb, higher education and vaccination were protective; advanced age, rural residence, chronic comorbidities, and non-medical occupations were risk factors. For HBcAb, advanced age, chronic comorbidities, and HBV exposure were risk factors, while higher education and vaccination were protective. **Conclusions:** Pregnant women in Nanning have a high HBV prevalence and insufficient immune protection. Targeted prevention strategies for reproductive-age women are urgently needed to reduce vertical transmission risk.

## 1. Introduction

Hepatitis B virus (HBV) infection is a critical global public health threat and a leading cause of chronic liver diseases, including liver cirrhosis and primary hepatocellular carcinoma (HCC). Epidemiological disparities in HBV endemicity are evident worldwide: the carrier rate remains low at 0.1–2.0% in the United States and Western Europe, whereas most countries in Africa and Asia sustain a substantially higher prevalence of 8–20% [1]. According to the World Health Organization (WHO), an estimated 254 million people globally suffered from chronic HBV infection in 2022, with 1.2 million new cases diagnosed annually. HBV-induced end-stage liver diseases, including liver cirrhosis and HCC, cause approximately 1.1 million deaths each year, over 85% of which occur in low- and middle-income countries, and 65% of these are concentrated in the African and Western Pacific regions, highlighting the disproportionate global disease burden in resource-limited settings [2,3].

Most of the global burden of chronic hepatitis B (CHB) is attributable to mother-to-child transmission (MTCT) occurring at or shortly after birth, which represents the dominant route of HBV transmission and accounts for approximately 90% of all primary HBV infections. Both perinatal and non-perinatal MTCT sustain viral circulation in populations and maintain long-term HBV endemicity worldwide. Notably, neonates infected with HBV during delivery have a 95% probability of progressing to lifelong chronic infection, and 15–40% of these chronically infected children will develop irreversible adult liver complications, including cirrhosis and HCC [4,5,6]. Given the pivotal role of MTCT in sustaining the global HBV epidemic, WHO has prioritized MTCT prevention as one of five core strategies to achieve the 2030 goal of eliminating viral hepatitis as a major public health threat [7]. Considerable global progress has been achieved in blocking perinatal HBV transmission via universal infant HBV immunization, particularly timely administration of the hepatitis B birth dose, which has proven highly effective in reducing childhood HBV new infections.

China is a highly endemic country for HBV infection and bears the world’s most severe societal disease burden attributed to chronic hepatitis B, post-hepatic cirrhotic lesions, and primary hepatocellular carcinoma [8]. Prior to the widespread implementation of HBV vaccination, two national epidemiological surveys on viral hepatitis were conducted in China. Both surveys demonstrated equivalent hepatitis B surface antigen (HBsAg) positivity rates between children and adults, indicating that HBV infection acquired during the neonatal and childhood periods is the primary determinant of the high HBV endemicity in China [9]. Over the past three decades, China has steadily established and optimized a comprehensive intervention system to block HBV MTCT. In 1992, neonatal hepatitis B vaccination was incorporated into the national routine immunization program and implemented on a voluntary, self-paid basis [8]. In 2002, childhood hepatitis B vaccination was further integrated into the national Expanded Programme on Immunization (EPI) with full government funding and free vaccination coverage, which dramatically improved national vaccination coverage [10]. Since 2005, vaccination has been provided to all neonates completely free of charge nationwide; catch-up vaccination was subsequently extended to previously unvaccinated children and adolescents. In 2015, the integrated prevention of mother-to-child transmission (iPMTCT) program targeting HIV, syphilis, and hepatitis B achieved full nationwide coverage, elevating the HBV screening rate among pregnant women to over 99% [11]. Nationally, the three-dose hepatitis B vaccination coverage among newborns has remained above 95% in recent years and reached 99.6% according to the latest statistics, with a timely birth-dose coverage of 95.6%, far exceeding the global average [8]. Benefiting from continuous policy optimization and standardized pediatric immunization management, China’s overall HBV prevalence declined markedly from 9.67% in 1992 to 5.86% in 2020 [12]. In particular, the HBsAg positive rate among individuals aged 0–29 years has dropped to 0.3% [10,13,14]. These remarkable achievements have transformed China from a high-endemic to a moderate-endemic region, with the most prominent prevention effects observed in pediatric populations [7].

Despite remarkable progress in pediatric prevention, no curative therapies are currently available to completely eradicate persistent HBV infection, leaving a substantial population of chronically infected individuals in China. Nationwide, over 90 million people live with chronic HBV infection, causing more than 300,000 HBV-attributable deaths each year and accounting for nearly half of the global mortality burden of HBV-related diseases [15,16]. The HBV prevalence among pregnant women in China has not declined significantly. In 2020, the national HBV prevalence among pregnant women reached 6.64%, which was higher than that of the general population [17]. Moreover, HBV infection among pregnant women shows distinct regional disparities across China, with provincial prevalence rates ranging from 1.53% to 11.9% and uniformly higher endemic levels in southern versus northern regions [17]. These findings indicate that pregnant women remain a key high-risk population for HBV prevention, with persistent and heavy disease burden [17,18,19,20,21]. These epidemiological characteristics render China a key region for achieving the WHO’s global target of eliminating hepatitis B by 2030 [7]. Currently, the combined immunoprophylaxis regimen of the hepatitis B vaccine and hepatitis B immunoglobulin (HBIG) administered within 12 h of birth has been universally adopted for HBV MTCT blocking in China. However, high maternal HBV viral load is well recognized as the leading independent risk factor for immunoprophylaxis failure and residual vertical transmission [22,23,24]. To address this challenge, China issued the Action Plan for Eliminating Mother-to-Child Transmission of HIV, Syphilis, and Hepatitis B (2022–2025) in 2022, which prioritizes HBV infection prevention among women of childbearing age to fundamentally reduce the risk of vertical transmission.

Guangxi Zhuang Autonomous Region is a high HBV-endemic province in southwestern China. Nanning, the regional capital, features dense, multi-ethnic, and highly mobile populations, contributing to sustained high local HBV prevalence. In 2020, the overall HBsAg positivity rate in Guangxi was 6.32%, with a substantially elevated rate of 9.84% among pregnant women [12,25]. In Nanning, the general HBsAg seroprevalence was 4.46%, whereas the prevalence among residents aged ≥15 years reached 9.60%, with the adult HBV infection burden markedly higher than that in minors [26]. The city’s reported hepatitis B incidence fluctuated between 71.84 and 126.53 per 100,000 population from 2015 to 2022, exhibiting an increasing temporal trend [27].

Supported by national standardized immunization and HBV mother-to-child transmission (MTCT) intervention programs, Nanning has achieved remarkable success in pediatric HBV prevention. Continuous surveillance data (2007–2019) demonstrated that full-course HBV vaccination coverage among 12-month-old infants remained steadily above 98%, and the timely 24-h postpartum vaccination rate exceeded 95%. A city-wide catch-up vaccination initiative targeting unvaccinated children and adolescents born in 1994–2001 yielded a coverage rate of over 95% [27]. Consequently, the HBsAg positivity rate among children aged <15 years in Nanning declined to 0.68% in 2018 [26]. Although marginally higher than the national average of 0.32%, this prevalence satisfies the WHO threshold (<1%) for pediatric HBV elimination.

In contrast to effective pediatric HBV control, adult HBV prevention in Nanning remains suboptimal, particularly among pregnant women, a pivotal high-risk group for HBV MTCT and persistent endemic transmission. Currently, adult HBV vaccination in Nanning adopts a voluntary, self-funded mode without mandatory immunization scheduling, resulting in prominent population immune deficiencies. Evidence shows that full-course HBV vaccination coverage among local medical students was merely 50.12% [28]. Despite a slight decreasing trend in recent years, the HBsAg prevalence among reproductive-age women in Guangxi remains as high as 9.84%, posing substantial challenges to regional HBV control.

Nevertheless, targeted epidemiological investigations focusing on HBV infection profiles and their determinants among pregnant women in Nanning are limited. Existing regional HBV prevention strategies lack localized refinement tailored to Nanning’s multi-ethnic aggregation and high population mobility characteristics. Previous Guangxi-based studies primarily focused on rural residents or broad age cohorts, with insufficient data on HBV serological status and immune profiles among urban–rural integrated pregnant populations. Furthermore, few studies have comprehensively explored the full spectrum of HBV serological markers, HBsAb geometric mean concentrations, and their associated factors in this specific population, creating notable research gaps.

We systematically analyzed the seroprevalence of five HBV markers, the HBV infection spectrum distribution, HBsAb-mediated immune protection levels, and independent influencing factors for HBV seropositivity among pregnant women in Nanning during 2024–2025. The findings will supplement local epidemiological data for pregnant populations, identify key high-risk factors and preventive weaknesses, and provide evidence-based references for optimizing refined regional HBV prevention and MTCT elimination strategies. This study is of clinical and public health significance for reducing regional HBV disease burden and facilitating the ultimate elimination of HBV perinatal transmission.

## 2. Materials and Methods

### 2.1. Setting and Participants

This study adopted a stratified cluster random sampling design to recruit pregnant women receiving prenatal care from October 2024 to May 2025 in Nanning, China. Five representative medical institutions covering urban, suburban, and rural areas were selected as survey sites, including the First People’s Hospital of Nanning in Qingxiu District, the Second People’s Hospital of Nanning in Jiangnan District, Binyang Maternal and Child Health Hospital, Longan County People’s Hospital, and Yongning District People’s Hospital. Inclusion criteria were: (1) having undergone prenatal examinations at these institutions from October 2024 to May 2025; (2) maintaining a maternal health management record; (3) current residence in Nanning; (4) providing informed consent and cooperating with the survey. Exclusion criteria were: (1) the presence of severe underlying conditions such as significant hepatic or renal dysfunction or malignant tumors; (2) cognitive impairment preventing questionnaire completion; (3) refusal of blood sample collection. The required sample size was calculated based on the formula $\;n=Z^{2}P(1-P)/d^{2}$, with a significance level of α = 0.05 (Z = 1.96), an expected positive rate P = 9.84% (source: regional Guangxi maternal surveillance report [12,25]), and an absolute permissible error d = 0.1 (±10% point estimate margin). A 10% dropout rate was accounted for in the sample size adjustment. Variance inflation resulting from cluster sampling was corrected using the design-effect formula: DEFF=1 + (m−1)ICC, where m denotes the average number of pregnant women recruited per medical institution. A conservative intra-cluster correlation coefficient (ICC=0.10) derived from relevant epidemiological studies was adopted to inflate the sample size and maintain the target precision.

### 2.2. Survey Content and Field Investigation

A self-designed structured questionnaire was used to collect participant information through face-to-face interviews conducted by uniformly trained investigators. The questionnaire covered three main dimensions: sociodemographic characteristics (age, residential location, ethnicity, occupation, educational level, and monthly household income per capita); clinical/behavioural history (common chronic comorbidities (defined as chronic hypertension, type 1/2 diabetes mellitus, chronic nephropathy, chronic liver disease, autoimmune disorders, and long-term cardiovascular disease, etc.), surgical history, blood transfusion history, acupuncture exposure, syringe sharing, and shared daily necessities); and HBV-related characteristics (HBV vaccination history, vaccination interval, and daily exposure to HBV patients or carriers). All questionnaires were checked immediately after completion to supplement missing information and correct logical errors, ensuring high data quality. In addition, hepatitis B vaccination records of the pregnant women since 2008 were verified through the Guangxi Immunization Planning Information Management System to cross-check self-reported vaccination history and minimize recall bias.

### 2.3. Specimen Collection and Laboratory Detection

Venous blood samples of 3–4 mL were collected from each participant and placed in non-anticoagulant tubes. After standing at room temperature for 30 min, the blood samples were centrifuged at 3000 rpm for 10 min to separate the serum. The isolated serum specimens were stored at −80 °C until detection. All samples were transported to the clinical laboratory of the Second People’s Hospital of Nanning via professional cold-chain vehicles for unified testing. Hepatitis B virus (HBV) serological markers (HBsAg, Anti-HBs, HBeAg, Anti-HBe, and Anti-HBc) were quantitatively detected using a fully automated chemiluminescence immunoassay analyzer with the magnetic particle chemiluminescence reagent kits manufactured by Zhengzhou Autobio Engineering Co., Ltd, Zhengzhou City, China. The analytical sensitivities were as follows: HBsAg 0.02 IU/mL, Anti-HBs 1 mIU/mL, HBeAg 0.03 IU/mL, Anti-HBe 0.06 PEIU/mL, and Anti-HBc 0.4 PEIU/mL. The intra-assay coefficients of variation (CVs) ranged from 2.1% to 6.4%, and the inter-assay CVs ranged from 8.4% to 14.8%. Prior to each batch of testing, the system was calibrated using the matching calibrators, and internal quality control (IQC) was performed using low-, medium-, and high-concentration quality control materials. External quality assessment (EQA) was conducted by the National Center for Clinical Laboratories (NCCL) and the Guangxi Center for Clinical Laboratory to continuously verify and maintain the accuracy of the laboratory testing. All experimental procedures strictly complied with official reagent package inserts and standardized laboratory standard operating procedures (SOPs). Uniform positive cutoff thresholds were predefined: HBsAg > 0.05 IU/mL, HBsAb > 10 mIU/mL, HBeAg > 0.1 IU/mL, HBeAb > 0.15 PEIU/mL, and HBcAb > 0.7 PEIU/mL.

### 2.4. Classification of HBV Infection Status

Participants were stratified into uninfected and HBV-infected groups according to composite five-marker serological profiles. Uninfected individuals included susceptible subjects (all five markers negative) and effectively immune subjects (isolated HBsAb seropositivity). Infected cases were further subdivided into four categories: previous or occult HBV infection (HBsAg-negative with positive core or e-antibodies), isolated HBsAg carriage (only HBsAg positive), inactive HBV infection (HBsAg-positive, HBeAb-positive, and HBcAb-positive, i.e., “small triple positive”), and active HBV infection (HBsAg-positive, HBeAg-positive, and HBcAb-positive, i.e., “large triple positive”). The detailed classification criteria based on HBV serological markers are shown in Table 1.

### 2.5. Statistical Analyses

Questionnaire data were double-entered and cross-checked using WPS Office 2019 (version 11.1.0.7881). Missing data were assumed to be missing-at-random (MAR). Seropositivity rates and their 95% confidence intervals were calculated using the Wilson-score method, and the chi-square test or Fisher’s exact test was used for between-group comparisons as appropriate. A multivariable logistic regression model was constructed using the stats package in R to screen for independent influencing factors associated with each of the five hepatitis B serological markers. For variable selection, covariates with *p* < 0.10 in univariate analyses and potential confounding variables predefined based on clinical considerations were included in the initial multivariable model. The variance inflation factor (VIF) was calculated via the car package (version 3.1.2) to detect multicollinearity among candidate variables; a VIF > 5 was defined as indicative of substantial multicollinearity, and variables exhibiting multicollinearity or lacking statistical contribution were excluded. The Hosmer–Lemeshow goodness-of-fit test was adopted to assess model calibration, with *p* > 0.05 indicating acceptable model fit. The area under the receiver-operating-characteristic curve (AUC) was calculated using the pROC package (version 1.18.5) to evaluate model discrimination performance, and AUC values ranging from 0.70 to 0.90 were interpreted as moderate-to-good predictive ability. The Shapiro–Wilk test confirmed that the log_10_-transformed data conformed to a normal distribution (*p* > 0.05). A generalized linear mixed-effects model (GLMM) (lme4 package (version 1.1.35)) was fitted to estimate geometric mean concentrations (GMCs) and geometric mean ratios (GMRs). Survey hospitals were incorporated into the model as random-intercept terms to adjust for intra-cluster correlation arising from the multicenter sampling design. This model was adjusted for confounding factors, including age, residential location, chronic underlying diseases, and hepatitis B vaccination status. All statistical analyses were performed using R software (version 4.5.1). A two-sided *p*-value < 0.05 was considered statistically significant.

## 3. Results

### 3.1. Baseline Characteristics of Study Participants

**Figure 1:**
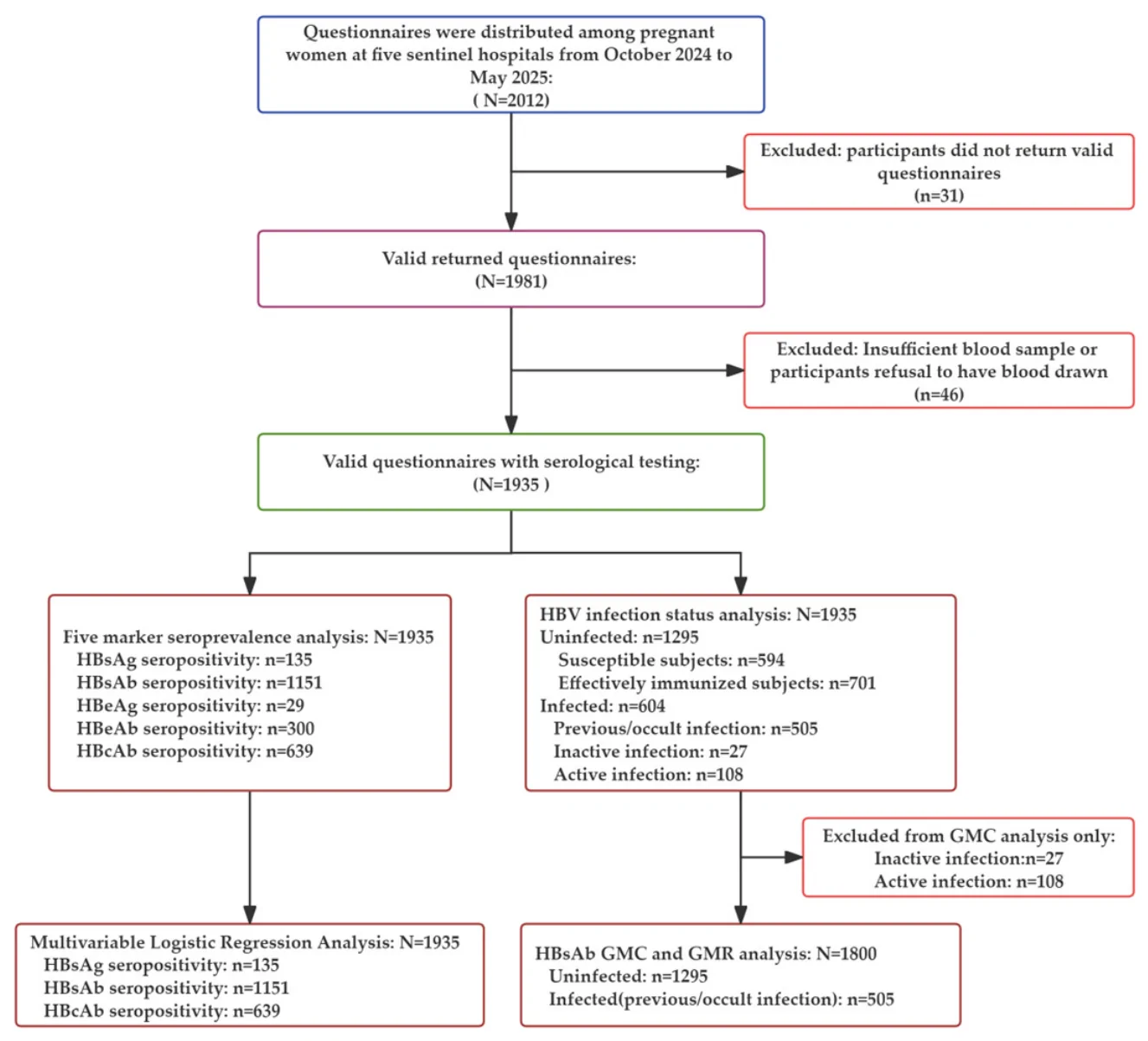
Flow chart of participant recruitment and enrollment of pregnant women in Nanning, 2024–2025.

A total of 2012 pregnant women were initially screened for study eligibility. Of these, 31 pregnant women did not return valid questionnaires, and 46 were excluded due to refusal of blood collection or unqualified blood samples (Figure 1). The final valid sample included 1935 pregnant women, yielding an effective response rate of 96.17%. The mean age of the enrolled pregnant women was 31.15 ± 5.45 years (range 14–47 years). The detailed distributions of sociodemographic characteristics (age group, residence, ethnicity, occupation, educational level, household per capita income), health status, HBV vaccination history, and parity are summarized in Table S1. Briefly, 44.13% of pregnant women resided in urban areas, 40.10% in county/township regions, and 15.76% in rural villages; ethnic composition comprised 51.78% Han, 45.06% Zhuang, and 3.15% other ethnic minorities. Self-employment represented the dominant occupational category (41.81%). 2.43% of pregnant women had chronic comorbidities. 12.25% had never received HBV vaccination, 5.48% completed their final vaccine dose within five years prior to the survey, 60.16% were vaccinated five or more years earlier, and 22.12% could not recall or provide a clear vaccination history. Full stratified constituent ratios across all sociodemographic, clinical, and immunization variables are summarized in Table 2.

### 3.2. Stratified Age Distribution of Composite HBV Infection Status

According to the comprehensive serological profiles of the five HBV markers (HBsAg, HBsAb, HBeAg, HBeAb, and HBcAb), 66.93% (1295/1935) of all pregnant women were classified as HBV-uninfected, and the remaining 33.07% (640/1935) as HBV-infected. Among uninfected individuals, 30.70% (594/1935) were susceptible with all serological markers negative, and 36.23% (701/1935) achieved an effective immune status with isolated HBsAb seropositivity. Among infected pregnant women, 26.10% (505/1935) had previous or occult HBV infection, 1.40% (27/1935) had inactive HBV infection (small triple positive), and 5.58% (108/1935) had active HBV infection (large triple positive). Age-stratified analysis revealed a distinct trend in HBV infection status. The proportion of uninfected individuals gradually decreased with advancing age, while the proportion of HBV-infected individuals increased progressively with age, and this trend difference was statistically significant (*p* < 0.001) (Table 3).

### 3.3. Seroprevalence of Five HBV Serological Markers

#### 3.3.1. Overall Seropositivity

Among the 1935 pregnant women, overall seropositivity rates (with 95% CI) for HBsAg, HBsAb, HBeAg, HBeAb, and HBcAb were 6.98% (5.92–8.20%), 59.48% (57.28–61.65%), 1.50% (1.05–2.14%), 15.50% (13.96–17.18%), and 33.02% (30.96–35.15%), respectively (Table 4).

#### 3.3.2. Serological Marker Distribution Stratified by Sociodemographic Factors

We observed distinct age gradients for HBsAg, HBeAb, and HBcAb seropositivity, with prevalence rising consistently from the youngest (14–24 years) to the oldest (40+ years) age group. HBsAb seropositivity initially increased, then stabilized, whereas HBeAg positivity showed no statistically significant age-related variation (*p* > 0.05). Pronounced urban–rural disparities were detected: rural pregnant women had significantly higher HBsAg and HBeAb seropositivity alongside markedly lower HBsAb protection compared with urban and county/township residents (all *p* < 0.01). Occupational status was strongly correlated with HBsAb seroprotection: medical staff maintained the highest HBsAb positive rate (82.03%), whereas farmers exhibited the lowest seroprotection rate (45.38%). Higher educational level attainment correlated positively with HBsAb seropositivity and negatively with HBeAb/HBcAb prevalence. Notably, pregnant women with a master’s or doctoral degree displayed an HBeAg positivity rate of 0% and an HBeAb positivity rate of 3.03% (1.31–14.55%). No significant ethnic differences were identified for any HBV serological marker (all *p* > 0.05), although the “other ethnicities” subgroup showed a relatively low HBsAg seropositivity of 1.64% (0.54–8.47%) (Table 4).

#### 3.3.3. Stratification by Vaccination History, Obstetric History, and Comorbidities

HBV vaccination status was the strongest modifiable correlate of all five serological markers. Pregnant women without vaccination history or with unknown immunization records displayed substantially elevated HBV infection marker prevalence and diminished HBsAb seroprotection relative to recently vaccinated pregnant women (dose interval < 5 years). Those vaccinated ≥5 years earlier demonstrated intermediate infection risk and lower antibody concentrations (all inter-group *p* < 0.001). Gravidity (≥3 births) correlated with elevated HBeAb and HBcAb seropositivity (*p* < 0.001), whereas gravidity showed no significant association with HBsAg, HBsAb, or HBeAg status. Pregnant women with chronic underlying diseases exhibited significantly higher rates of all HBV infection markers compared with pregnant women without comorbidities (all *p* < 0.05) (Table 5).

#### 3.3.4. Serological Profiles Stratified by HBV Exposure History

Close daily contact with confirmed HBV carriers or chronically infected patients was associated with significantly elevated HBsAg, HBeAg, HBeAb, and HBcAb seropositivity (all *p* < 0.001). Self-reported syringe sharing correlated with a higher prevalence of HBsAg and HBeAb (*p* < 0.001 and *p* = 0.027, respectively) (Table 5). No statistically significant differences in five-marker seroprevalence were observed for surgical history, blood transfusion exposure, acupuncture treatment, or shared daily tableware supplies (all *p* > 0.05) (Table S1).

### 3.4. Independent Influencing Factors for HBsAg, HBsAb, and HBcAb Seropositivity

#### 3.4.1. Correlates of HBsAg Seropositivity

Multivariate logistic regression analysis demonstrated that advanced age, history of sharing syringes, chronic comorbidities, unvaccinated status, and HBV infection exposure were independent risk factors for HBsAg positivity. Compared with the 14– year group, the 25–, 30–, 35–, and 40– year groups presented significantly higher HBsAg positivity risks, with ORs (95% CIs) of 2.02 (0.80–5.10), 5.34 (2.19–13.02), 6.24 (2.50–15.59), and 11.26 (4.09–31.02), respectively. Compared with pregnant women who were unvaccinated, those with vaccination intervals of <5 years and ≥5 years had lower HBsAg positivity, with ORs (95% CIs) of 0.06 (0.01–0.42) and 0.26 (0.16–0.43), respectively. Relative to pregnant women with surrounding HBV exposure, those with no exposure or unclear exposure status showed reduced HBsAg risk, with ORs (95% CIs) of 0.12 (0.07–0.19) and 0.27 (0.15–0.46), respectively (Table 6). Coding specifications for variables included in the multivariate logistic regression model are provided in Table S2.

#### 3.4.2. Correlates of HBsAb Seropositivity

Multivariate logistic regression analysis revealed that advanced age, urban residence, higher educational level, medical occupation, absence of chronic comorbidities, and a valid hepatitis B vaccination history were identified as protective factors for HBsAb seropositivity. Compared with the 14–year group, the 25–, 30–, 35–, and 40–year groups had higher probabilities of HBsAb seropositivity, with ORs (95% CIs) of 1.79 (1.31–2.46), 1.51 (1.10–2.08), 2.00 (1.41–2.84), and 2.17 (1.32–3.57), respectively. In comparison with urban residents, pregnant women living in county/township and rural areas had lower HBsAb seropositivity, with ORs (95% CIs) of 0.74 (0.56–0.97) and 0.60 (0.43–0.86). Regarding educational level, pregnant women with a bachelor’s degree and postgraduate education had higher HBsAb seropositivity than those with junior high school education or below, with ORs (95% CIs) of 1.85 (1.38–2.49) and 4.16 (1.53–11.30), respectively. Taking medical staff as the reference group, personnel in public institutions, commercial service workers, factory workers, self-employed individuals, unemployed individuals, and farmers exhibited lower HBsAb seropositivity, with ORs (95% CIs) of 0.38 (0.19–0.76), 0.41 (0.23–0.74), 0.30 (0.13–0.67), 0.41 (0.25–0.68), 0.37 (0.20–0.67), and 0.24 (0.13–0.44), respectively. Pregnant women without chronic comorbidities were more likely to be HBsAb-positive (OR = 2.24, 95% CI: 1.20–4.17) than those with underlying diseases. Compared with pregnant women who were unvaccinated, those with vaccination intervals of <5 years and ≥5 years had significantly higher HBsAb seropositivity, with ORs (95% CIs) of 23.29 (9.09–59.66) and 1.86 (1.39–2.48) (Table 7).

#### 3.4.3. Correlates of HBcAb Seropositivity

Multivariate logistic regression analysis indicated that advanced age was an independent risk factor for HBcAb positivity, while higher educational level, absence of chronic comorbidities, a short vaccination interval (<5 years), and no surrounding HBV exposure were protective factors. Compared with the 14– year group, the 25–, 30–, 35–, and 40– year groups showed progressively increased HBcAb-positive risks, with ORs (95% CIs) of 2.03 (1.32–3.12), 4.18 (2.73–6.41), 5.93 (3.80–9.25), and 12.32 (7.00–21.68), respectively, presenting a significant age-dependent rising trend. Pregnant women with a bachelor’s degree and postgraduate education had lower HBcAb positivity than those with junior high school education or below, with ORs (95% CIs) of 0.67 (0.49–0.91) and 0.36 (0.15–0.90), respectively. The absence of chronic comorbidities reduced the HBcAb-positive risk (OR = 0.29, 95% CI: 0.16–0.55). Pregnant women vaccinated within 5 years had a lower HBcAb positivity rate than those who were unvaccinated (OR = 0.24, 95% CI: 0.12–0.45). Relative to pregnant women with no surrounding HBV exposure, those with exposure or unclear exposure status had higher HBcAb positivity, with ORs (95% CIs) of 2.61 (1.93–3.52) and 1.18 (0.91–1.53), respectively (Table 8).

### 3.5. Stratified HBsAb Geometric Mean Concentrations

The geometric mean concentration (GMC) of HBsAb was analyzed among uninfected pregnant women and those with previous/occult HBV infection. For the total 1800 eligible pregnant women, the overall GMC of HBsAb was 11.22 (95% CI: 9.50–13.20) mIU/mL. Stratified by infection status, the GMC was 4.63 (95% CI: 3.72–5.75) mIU/mL among 1295 uninfected pregnant women and 109.48 (95% CI: 88.22–135.85) mIU/mL among 505 pregnant women with previous/occult HBV infection. Pregnant women with previous or occult HBV infection exhibited a significantly higher HBsAb GMC than uninfected individuals (*p* < 0.001). Among uninfected pregnant women, the HBsAb GMC differed significantly across subgroups stratified by age, household type, occupation, educational level, household per capita monthly income, vaccination history, parity, and surrounding HBV exposure status (all *p* < 0.05). In contrast, no significant differences in HBsAb GMC were observed regarding ethnicity, chronic comorbidities, surgical history, blood transfusion history, acupuncture history, syringe sharing, and shared daily tableware supplies (all *p* > 0.05). For pregnant women with previous/occult HBV infection, significant differences in HBsAb GMC were only found in subgroups stratified by age and household per capita monthly income (both *p* < 0.05) (Table 9).

## 4. Discussion

This cross-sectional study systematically analyzed the current epidemiological status, immune protection level, and associated influencing factors of HBV infection among pregnant women in Nanning (2024–2025), providing regional evidence for optimizing maternal HBV prevention and blocking perinatal transmission in southwest China. The findings reveal a moderate HBV endemic burden among local childbearing women, prominent population and immune stratification, and prominent gaps between regional prevention practices and global and China elimination targets, which deliver targeted, actionable implications for maternal HBV prevention in high-burden multi-ethnic border regions.

The overall maternal HBsAg prevalence of 6.98% identified in Nanning reflects a moderate endemic status with distinct regional epidemic heterogeneity. Nationally, this rate exceeds the 2020 national maternal average (6.64%) and the 2020 general population level (5.86%) [13,17], and is substantially higher than low-endemic provincial regions including Jiangsu, Xinjiang, and Qingdao [29,30] though lower than high-burden coastal areas such as Quanzhou, Fujian [31]. Temporally, the local prevalence has decreased by 29.07% compared with Guangxi’s 2016 maternal data (9.84%), aligning with the nationwide declining epidemic trend and verifying the cumulative effectiveness of universal childhood immunization and standardized prenatal screening programs in China [12,25,32].

Global and Asian cross-regional comparison further clarifies Nanning’s epidemic positioning and elimination gaps. Globally, low-prevalence Western regions maintain maternal HBsAg prevalence below 0.5%, far below the local level [33]. Within Asia, Southeast Asian countries have achieved prominent epidemic declines through optimized preconception and rural vaccination strategies: Thailand reduced maternal HBV prevalence from 6.11% in 2003 to 3.15% in 2022, while Cambodia reports a maternal prevalence of 4.28% [34,35]. In contrast, Nanning’s residual maternal burden remains markedly higher than these similar middle-prevalence regions, indicating insufficient local intervention efficiency despite consistent downward trends. Most critically, this prevalence severely conflicts with WHO 2030 elimination requirements, which mandate under-5 children HBsAg prevalence <0.1% and MTCT rate <2% [36]. A persistently high maternal carrier rate constitutes the fundamental source of residual vertical transmission, rendering southwest border multi-ethnic regions a key restrictive barrier for national and global hepatitis B elimination. Serological ternary distribution (susceptible, immune, infected populations each accounting for one-third) further confirms a large-scale sustainable susceptible reservoir in local childbearing women, which continuously offsets the benefits of routine prenatal screening and poses a long-term public health challenge.

Serological pattern analysis demonstrated that uninfected susceptible individuals, effectively immunized individuals, and HBV-infected individuals each accounted for approximately one-third of the pregnant population in Nanning, which was consistent with findings from previous domestic studies [37]. Age-stratified analysis showed that younger pregnant women were predominantly susceptible or immune-protected, whereas older participants had substantially higher proportions of previous/occult and current HBV infections. This age-specific distribution indicates a persistent reservoir of HBV infection among local pregnant women, leading to a potential high risk of mother-to-child transmission (MTCT) and posing a substantial challenge to China’s progress toward the WHO global goal of eliminating viral hepatitis.

This study found that HBV infection among pregnant women exhibits distinct age distribution patterns, with the HBsAg positivity rate gradually increasing with age—from 2.45% in those aged 14–24 to 15.09% in those aged 40–47. As age increases, the proportions of susceptible individuals and those with a history of immunity progressively decline, while the proportions of previously infected individuals and current infections continue to rise. Multivariate regression analysis confirmed that advanced age is a risk factor for HBsAg seropositivity, consistent with findings from related domestic studies [4]. This is attributed not only to the natural decline of protective antibodies and the cumulative exposure to the virus but also closely linked to the implementation progress of China’s hepatitis B immunization program [4]. In this study, pregnant women under 25 years old were born after 2000; during this period, the Chinese government fully implemented the Expanded Program on Immunization (EPI), ensuring free hepatitis B vaccination for children, with a full vaccination rate exceeding 85% [10], effectively reducing HBV infection risks in this age group. In contrast, pregnant women aged 25 and above were predominantly born between 1980 and 2000, when the hepatitis B vaccine had just been introduced clinically and had not yet been widely incorporated into the national immunization program. Children in this group had to pay out-of-pocket for vaccination, resulting in low overall vaccination rates and limited active immune protection, with most obtaining immunity through natural infection. Additionally, this demographic exhibited broader social exposure and more pregnancies, further increasing HBV exposure risks [10], leading to a dual-high phenomenon in both HBV infection rates and HBsAb seropositivity rates among this birth cohort. Age distribution analysis reveals the 40+ age group has a 15.09% HBsAg prevalence, six times higher than the 14–24 age group. This aligns with national data showing 1995-born populations have 3.2 times higher HBsAg prevalence than post-1995 cohorts, highlighting persistent immunity gaps among individuals born before universal hepatitis B vaccination implementation. This provides direct evidence supporting national strategies to “prioritize filling immunity gaps in populations born before 1995.”

National studies have documented significant regional disparities in maternal HBsAg prevalence across China, with higher rates in southern and western provinces and a persistent urban–rural gap [17,21]. This study found that the HBsAg-positive risk among rural pregnant women was 1.62 times that among urban residents, while their HBsAb aGMR was only 0.25 of the urban level, This spatial distribution pattern is consistent with the national and regional epidemiological characteristics of HBV in Guangxi [17,38,39]. The underlying reasons may involve uneven allocation of medical and health resources, inconsistent implementation of prenatal HBV screening, and disparities in vaccination coverage and health awareness between urban and rural populations. Notably, no significant ethnic differences were observed in HBV serological markers or protective antibody levels, indicating that traditional ethnic customs no longer serve as a major determinant of HBV infection among local pregnant women following the continuous integration of lifestyles across different ethnic groups. Among uninfected pregnant women, medical workers and individuals with higher educational levels maintained higher HBsAb positive rates and stronger antibody protection. This phenomenon is likely attributed to their superior knowledge of HBV prevention, stronger health awareness, higher vaccination adherence, and more standardized booster immunization practices, which collectively sustain stable immune protection. These findings amplify national trends and underscore the urgency of prioritizing resource allocation to grassroots, rural, and remote areas as mandated by the China Viral Hepatitis Prevention and Control Action Plan (2025–2030) [40].

From the perspective of population immunity, the overall vaccination coverage and long-term immune maintenance among reproductive-age women in Nanning remain suboptimal. In the present cohort, only 65.63% of pregnant women had a documented hepatitis B vaccination history, and merely 5.48% completed vaccination within the past five years, suggesting insufficient primary vaccination coverage and inadequate recent booster immunization among local women of childbearing age. In terms of immune response quality, pregnant women vaccinated within five years achieved an HBsAb seroconversion rate of 95.28% and a high geometric mean concentration (GMC) of 177.30 (95% CI: 48.06–654.11) mIU/mL, indicating robust protective immunity. In contrast, unvaccinated pregnant women had a seroconversion rate of only 46.8% and an extremely low HBsAb GMC of 0.94 (95% CI: 0.27–3.28) mIU/mL, representing negligible immune protection. These results strongly verify that standardized hepatitis B vaccination effectively enhances immune protection in reproductive-age females. Among vaccinated individuals, those with an interval of ≥5 years since the last vaccination had a breakthrough infection rate of 4.30%, which was 4.57-fold higher than that in women vaccinated within five years. Most breakthrough infections occurred in pregnant women over 25 years of age with a vaccination interval exceeding 20 years. Accumulated evidence indicates that fully vaccinated immunocompetent individuals can maintain protective immunity for approximately 20 years, but antibody titers decline continuously over time, and childhood vaccination induces faster antibody attenuation, which frequently drops below the protective threshold of 10 mIU/mL in adulthood [4]. Accordingly, prolonged protective antibody attenuation is a significant factor contributing to breakthrough infections among older pregnant women. Standardized hepatitis B vaccination remains the most cost-effective strategy for preventing maternal HBV infection and blocking vertical transmission. The 2024 WHO guidelines emphasize universal HBV screening for pregnant women, standardized MTCT prophylaxis, and targeted immune intervention for susceptible populations to accelerate global hepatitis B elimination [2]. Notably, WHO standard protocols state that routine booster vaccination is generally not required for fully immunized individuals with documented long-term immune protection, while serological surveillance and supplementary vaccination are recommended for individuals with insufficient HBsAb titers, particularly in high-endemic regions [41]. Consistent with WHO propositions, updated Chinese national hepatitis B prevention guidelines also advocate pre-pregnancy HBsAb screening and individualized supplementary immunization for reproductive-age females with inadequate immune protection, rather than universal booster administration [2,3,42]. This study also identified that pregnant women with chronic underlying diseases or a history of close HBV exposure were high-risk populations for HBV infection. Existing studies have demonstrated that comorbid HBV infection can exacerbate pre-existing chronic conditions and significantly increase the long-term risks of liver cirrhosis and hepatocellular carcinoma. For instance, patients with concurrent diabetes and HBV infection have a 2–3 fold higher risk of severe liver injury, cirrhosis, and primary liver cancer compared with the general population [43]. Therefore, targeted strategies focusing on high-risk pregnant women with underlying diseases or definite HBV exposure are urgently required, including rigorous pre-pregnancy and prenatal HBV serological screening, timely vaccination for susceptible individuals, and standardized follow-up and treatment for confirmed HBV cases to minimize new infections and MTCT risks.

Several limitations of this study need to be objectively acknowledged. First, the cross-sectional design only supports the analysis of statistical associations between influencing factors and HBV infection/immune status, and cannot confirm causal relationships. Second, partial vaccination history information relied on retrospective self-report, which may introduce recall bias. Third, this study lacked HBV DNA viral load quantification and infant follow-up data, making it impossible to quantitatively evaluate actual MTCT risk and intervention efficacy. In addition, long-term dynamic antibody monitoring was not conducted, and detailed stratification of vaccination intervals beyond five years was absent, limiting the accuracy of long-term immune persistence assessment. Finally, the study was conducted in partial medical institutions of Nanning, and unmeasured confounding factors may exist, so the generalization of conclusions to other regions needs prudent verification.

In conclusion, this observational study demonstrates that the prevalence of HBV infection among pregnant women in Nanning is relatively higher than the national average, highlighting notable gaps in local maternal HBV prevention and control practices. The epidemiological findings indicate distinct urban–rural disparities in HBV endemicity, elevated infection prevalence among older pregnant women and those with chronic comorbidities, suboptimal vaccination coverage in reproductive-age females, and prevalent age-related attenuation of protective HBsAb levels in this population. These observed associations underscore the need for targeted, refined optimization of regional HBV prevention strategies for childbearing women, rather than confirming causal relationships between the examined factors and HBV epidemic status. Current global and domestic guidelines provide evolving evidence for optimized population-based HBV immunization management. Collectively, this study’s findings reinforce the public health significance of precise maternal HBV immune management in high-burden areas. Integrating routine pre-pregnancy HBsAb detection into standardized prenatal screening programs and implementing individualized supplementary vaccination for antibody-deficient women can effectively consolidate population immune barriers among reproductive-age females. Targeted intervention focusing on rural populations, older pregnant women, and individuals with chronic comorbidities is conducive to narrowing urban–rural epidemic inequalities, reducing regional HBV transmission risk, and supporting the implementation of national and global strategies for hepatitis B elimination.

## Figures and Tables

**Table 1:** Definition of HBV Infection Status.

HBV Infection Status	HBsAg	HBsAb	HBeAg	HBeAb	HBcAb
Uninfected					
Susceptible subjects	−	−	−	−	−
Effectively immunized subjects	−	+	−	−	−
Infected					
Previous/occult infection	−	+/−	−	+/−	+
Inactive infection	+	−	−	+/−	+
Active infection	+	−	+	−	+/−

Note: +, positive; −, negative; +/−, positive or negative.

**Table 2:** Basic characteristics of study participants (N = 1935).

Characteristics	N	Percentage (%)
Age (years)		
14–	245	12.66
25–	584	30.18
30–	614	31.73
35–	386	19.95
40–	106	5.48
Household type		
Urban	854	44.13
County/township	776	40.10
Rural	305	15.76
Ethnicity		
Han	1002	51.78
Zhuang	872	45.06
Others	61	3.15
Occupation		
Medical staff	128	6.61
Teacher	111	5.74
Enterprise employees	205	10.59
Institutional staff	77	3.98
Commercial service staff	151	7.80
Worker	42	2.17
Self-employed	809	41.81
Housework/unemployed	126	6.51
Farmer	130	6.72
Others	156	8.06
Educational level		
Junior high school and below	535	27.65
High school/vocational school	393	20.31
Junior college	453	23.41
Bachelor’s degree	521	26.93
Master’s/Doctoral degree	33	1.71
Monthly household income per capita (CNY)		
<2000	400	20.67
2000–	1093	56.49
5000–	351	18.14
10,000–	91	4.70
Comorbidity		
Yes	47	2.43
No	1888	97.57
Vaccination history		
Unvaccinated	237	12.25
Interval < 5 years	106	5.48
Interval ≥ 5 years	1164	60.16
Unknown	428	22.12
Gravidity		
G1	833	43.05
G2	647	33.44
G3 or more	455	23.51

**Table 3:** Age-specific distribution of HBV infection status among pregnant women in Nanning, 2024–2025.

HBV Infection Status	Age Group (Years)					Total	
	14–	25–	30–	35–	40–	N	Proportion (%)
Uninfected	213	452	387	205	38	1295	66.93
Susceptible subjects	125	186	181	87	15	594	30.70
Effectively immunized subjects	88	266	206	118	23	701	36.23
Infected	32	132	227	181	68	640	33.07
Previous/occult infection	26	109	177	141	52	505	26.10
Inactive infection	4	5	11	5	2	27	1.40
Active infection	2	18	39	35	14	108	5.58
Total	245	584	614	386	106	1935	100.00

**Table 4:** Distribution of HBV Serological Markers by Sociodemographic Characteristics Among Pregnant Women in Nanning City, 2024–2025.

Var	N	HBsAg			HBsAb			HBeAg			HBeAb			HBcAb		
		*n*	Rate% (95% CI)	*p*	*n*	Rate % (95% CI)	*p*	*n*	Rate% (95% CI)	*p*	*n*	Rate % (95% CI)	*p*	*n*	Rate % (95% CI)	*p*
Total	1935	135	6.98 (5.92–8.20)		1151	59.48 (57.28–61.65)		29	1.50 (1.05–2.14)		300	15.50 (13.96–17.18)		639	33.02 (30.96–35.15)	
Age (years)																
14–	245	6	2.45 (1.16–5.21)	ref	106	43.27 (37.31–49.43)	ref	4	1.63 (0.66–4.10) *	ref	9	3.67 (1.98–6.80)	ref	31	12.65 (9.12–17.34)	ref
25–	584	23	3.94 (2.65–5.83)	0.291	363	62.16 (58.18–65.97)	<0.001	6	1.03 (0.48–2.22)	0.47	54	9.25 (7.17–11.85)	0.008	132	22.6 (19.42–26.15)	0.001
30–	614	50	8.14 (6.24–10.56)	0.004	368	59.93 (56.03–63.71)	<0.001	11	1.79 (1.01–3.17)	0.873	106	17.26 (14.50–20.44)	<0.001	227	36.97 (33.27–40.84)	<0.001
35–	386	40	10.36 (7.73–13.77)	0.001	245	63.47 (58.6–68.07)	<0.001	6	1.55 (0.73–3.34)	0.939	90	23.32 (19.41–27.74)	<0.001	181	46.89 (42.02–51.83)	<0.001
40–	106	16	15.09 (9.75–22.88)	<0.001	69	65.09 (55.95–73.18)	<0.001	2	1.89 (0.63–6.51) *	0.866	41	38.68 (30.28–47.87)	<0.001	68	64.15 (54.99–72.32)	<0.001
Household type																
Urban	854	49	5.74 (4.37–7.50)	ref	567	66.39 (63.17–69.47)	ref	14	1.64 (0.98–2.73)	ref	116	13.58 (11.46–16.03)	ref	274	32.08 (29.05–35.28)	ref
County/township	776	52	6.70 (5.16–8.67)	0.421	439	56.57 (53.08–60)	<0.001	10	1.29 (0.71–2.35)	0.558	118	15.21 (12.86–17.89)	0.351	252	32.47 (29.29–35.83)	0.866
Rural	305	34	11.15 (8.13–15.13)	0.002	145	47.54 (42.07–53.07)	<0.001	5	1.64 (0.72–3.76)	1	66	21.64 (17.44–26.54)	0.001	113	37.05 (31.89–42.53)	0.115
Ethnicity																
Han	1002	65	6.49 (5.13–8.18)	ref	604	60.28 (57.23–63.25)	ref	19	1.90 (1.22–2.94)	ref	145	14.47 (12.44–16.78)	ref	333	33.23 (30.4–36.2)	ref
Zhuang	872	69	7.91 (6.31–9.89)	0.233	508	58.26 (54.97–61.47)	0.374	9	1.03 (0.55–1.95)	0.13	146	16.74 (14.42–19.36)	0.176	292	33.49 (30.45–36.67)	0.908
Others	61	1	1.64 (0.54–8.47) *	0.161	39	63.93 (52.09–74.13)	0.571	1	1.64 (0.54–8.47) *	0.886	9	14.75 (8.49–25.2)	0.951	14	22.95 (14.81–34.3)	0.100
Occupation																
Medical staff	128	6	4.69 (2.28–9.74)	ref	105	82.03 (74.67–87.52)	ref	1	0.78 (0.20–4.23) *	ref	15	11.72 (7.39–18.27)	ref	40	31.25 (24.10–39.50)	ref
Teacher	111	4	3.60 (1.54–8.78) *	0.677	79	71.17 (62.43–78.5)	0.048	3	2.70 (1.04–7.53) *	0.278	13	11.71 (7.17–18.81)	0.999	34	30.63 (23.11–39.45)	0.918
Enterprise employees	205	12	5.85 (3.44–9.89)	0.648	137	66.83 (60.24–72.80)	0.003	2	0.98 (0.30–3.46) *	0.855	25	12.20 (8.48–17.3)	0.897	58	28.29 (22.68–34.70)	0.565
Institutional staff	77	7	9.09 (4.79–17.28)	0.218	49	63.64 (52.98–73.00)	0.004	1	1.30 (0.39–6.84) *	0.718	14	18.18 (11.56–27.83)	0.202	26	33.77 (24.69–44.38)	0.709
Commercial service staff	151	11	7.28 (4.22–12.47)	0.37	91	60.26 (52.49–67.53)	<0.001	2	1.32 (0.42–4.65) *	0.665	26	17.22 (12.18–23.88)	0.198	59	39.07 (31.84–46.84)	0.174
Worker	42	5	11.90 (6.02–24.17)	0.111	24	57.14 (43.41–69.68)	0.001	2	4.76 (1.92–15.18) *	0.135	7	16.67 (9.25–29.67)	0.409	19	45.24 (32.43–58.84)	0.101
Self-employed	809	58	7.17 (5.60–9.15)	0.305	456	56.37 (52.94–59.73)	<0.001	12	1.48 (0.85–2.57)	0.535	131	16.19 (13.83–18.88)	0.197	268	33.13 (29.99–36.43)	0.674
Housework/unemployed	126	7	5.56 (2.84–10.90)	0.754	67	53.17 (44.75–61.41)	<0.001	2	1.59 (0.51–5.53) *	0.56	19	15.08 (10.06–22.17)	0.433	37	29.37 (22.35–37.6)	0.744
Farmer	130	13	10.00 (6.09–16.21)	0.11	59	45.38 (37.32–53.71)	<0.001	1	0.77 (0.19–4.17) *	0.991	33	25.38 (18.9–33.29)	0.006	51	39.23 (31.5–47.58)	0.181
Others	156	12	7.69 (4.56–12.86)	0.306	84	53.85 (46.21–61.30)	<0.001	3	1.92 (0.71–5.44) *	0.432	17	10.9 (7.03–16.64)	0.828	47	30.13 (23.65–37.56)	0.838
Educational level																
Junior high school and below	535	47	8.79 (6.69–11.47)	ref	271	50.65 (46.46–54.84)	ref	9	1.68 (0.90–3.16)	ref	116	21.68 (18.42–25.34)	ref	214	40.00 (35.96–44.18)	ref
High school/vocational school	393	27	6.87 (4.79–9.79)	0.288	227	57.76 (52.87–62.50)	0.032	5	1.27 (0.56–2.93)	0.614	65	16.54 (13.23–20.5)	0.051	136	34.61 (30.12–39.39)	0.094
Junior college	453	33	7.28 (5.25–10.03)	0.390	259	57.17 (52.61–61.61)	0.041	9	1.99 (1.06–3.72)	0.722	56	12.36 (9.67–15.69)	<0.001	131	28.92 (24.97–33.22)	<0.001
Bachelor’s degree	521	28	5.37 (3.76–7.64)	0.033	366	70.25 (66.22–73.99)	<0.001	6	1.15 (0.54–2.48)	0.469	62	11.90 (9.42–14.94)	<0.001	151	28.98 (25.28–32.99)	<0.001
Master’s/Doctoral degree	33	0	0(0.54–9.88) *	0.973	28	84.85 (70.35–92.08)	0.001	0	0 *	0.984	1	3.03 (1.31–14.55) *	0.032	7	21.21 (12.09–36.34)	0.037
Monthly household income per capita (CNY)																
<2000	400	38	9.50 (7.03–12.74)	ref	208	52.00 (47.15–56.81)	ref	4	1.00 (0.40–2.53)	ref	77	19.25 (15.72–23.36)	ref	142	35.5 (31.01–40.26)	ref
2000–4999	1093	64	5.86 (4.62–7.40)	0.014	676	61.85 (58.94–64.67)	0.001	17	1.56 (0.98–2.47)	0.423	159	14.55 (12.59–16.75)	0.028	358	32.75 (30.05–35.58)	0.320
5000–9999	351	25	7.12 (4.90–10.27)	0.242	214	60.97 (55.83–65.87)	0.014	8	2.28 (1.18–4.41)	0.175	48	13.68 (10.51–17.63)	0.042	108	30.77 (26.22–35.73)	0.170
≥10,000	91	8	8.79 (4.76–16.16)	0.834	53	58.24 (48.38–67.44)	0.282	0	0 *	0.984	16	17.58 (11.44–26.35)	0.714	31	34.07 (25.54–43.88)	0.796

Note: * Statistical estimates with 95% CIs are unstable for sample sizes of n < 5.

**Table 5:** Distribution of HBV Serological Markers by Vaccination, Obstetric History, Comorbidities, and Exposure History Among Pregnant Women in Nanning City, 2024–2025.

Var	N	HBsAg			HBsAb			HBeAg			HBeAb			HBcAb		
		*n*	Rate% (95% CI)	*p*	*n*	Rate% (95% CI)	*p*	*n*	Rate% (95% CI)	*p*	*n*	Rate% (95% CI)	*p*	*n*	Rate% (95% CI)	*p*
Total	1935	135	6.98 (5.92–8.20)		1151	59.48 (57.28–61.65)		29	1.50 (1.05–2.14)		300	15.50 (13.96–17.18)		639	33.02 (30.96–35.15)	
Comorbidity																
Yes	47	18	38.30 (26.80–51.56)	ref	20	42.55 (30.54–55.69)	ref	6	12.77 (6.71–24.45)	ref	16	34.04 (23.16–47.34)	ref	30	63.83 (50.54–75.03)	ref
No	1888	117	6.20 (5.20–7.37)	<0.001	1131	59.9 (57.68–62.09)	0.019	23	1.22 (0.81–1.82)	<0.001	284	15.04 (13.50–16.72)	0.001	609	32.26 (30.19–34.39)	<0.001
Vaccination history																
Unvaccinated	237	32	13.5 (9.80–18.37)	ref	111	46.84 (40.68–53.09)	ref	10	4.22 (2.35–7.55)	ref	51	21.52 (16.85–27.10)	ref	89	37.55 (31.73–43.77)	ref
Interval <5 years	106	1	0.94 (0.25–5.06) *	0.006	101	95.28 (89.58–97.82)	<0.001	0	0 (0.06–3.44) *	0.987	6	5.66 (2.78–11.64)	0.001	14	13.21 (8.26–20.73)	<0.001
Interval ≥5 years	1164	50	4.30 (3.28–5.61)	<0.001	738	63.40 (60.60–66.11)	<0.001	10	0.86 (0.47–1.57)	<0.001	167	14.35 (12.46–16.47)	0.006	385	33.08 (30.44–35.82)	0.185
Unknown	428	52	12.15 (9.41–15.56)	0.615	201	46.96 (42.32–51.65)	0.975	9	2.10 (1.12–3.93)	0.124	76	17.76 (14.46–21.63)	0.238	151	35.28 (30.94–39.88)	0.559
Gravidity																
G1	833	57	6.84 (5.33–8.75)	ref	492	59.06 (55.71–62.34)	ref	16	1.92 (1.19–3.09)	ref	111	13.33 (11.20–15.79)	ref	229	27.49 (24.58–30.61)	ref
G2	647	44	6.80 (5.12–8.99)	0.975	384	59.35 (55.54–63.05)	0.911	10	1.55 (0.85–2.82)	0.587	89	13.76 (11.33–16.61)	0.810	213	32.92 (29.43–36.61)	0.024
G3 or more	455	34	7.47 (5.42–10.24)	0.673	275	60.44 (55.91–64.79)	0.631	3	0.66 (0.23–1.91) *	0.087	100	21.98 (18.45–25.98)	0	197	43.30 (38.86–47.85)	<0.001
Syringe sharing history																
No	1838	120	6.53 (5.49–7.75)	ref	1100	59.85 (57.59–62.06)	ref	27	1.47 (1.01–2.13)	ref	276	15.02 (13.46–16.72)	ref	597	32.48 (30.38–34.65)	ref
Yes	9	3	33.33 (19.91–56.72) *	0.006	4	44.44 (27.02–65.19) *	0.355	0	0 *	0.988	3	33.33 (19.91–56.72) *	0.143	5	55.56 (34.81–72.98)	0.156
Unknown	88	12	13.64 (8.28–22.04)	0.012	47	53.41 (43.49–63.04)	0.23	2	2.27 (0.78–7.76) *	0.549	21	23.86 (16.54–33.38)	0.027	37	42.05 (32.7–52.06)	0.064
HBV patients/infected persons around																
No	1274	47	3.69 (2.79–4.87)	ref	767	60.20 (57.5–62.85)	ref	6	0.47 (0.22–1.02)	ref	160	12.56 (10.86–14.49)	ref	375	29.43 (27–31.99)	ref
Yes	251	55	21.91 (17.32–27.36)	<0.001	155	61.75 (55.7–67.46)	0.646	19	7.57 (4.95–11.47)	<0.001	72	28.69 (23.53–34.49)	<0.001	134	53.39 (47.3–59.37)	<0.001
Unknown	410	33	8.05 (5.81–11.06)	<0.001	229	55.85 (51.06–60.54)	0.119	4	0.98 (0.39–2.47) *	0.258	68	16.59 (13.33–20.46)	0.039	130	31.71 (27.43–36.32)	0.383

Note: * Statistical estimates with 95% CIs are unstable for sample sizes of n < 5.

**Table 6:** Multivariate Logistic Regression Analysis of the Distribution of HBsAg Seropositivity Among Pregnant Women in Nanning City (2024–2025).

Variable	β	S.E.	aOR (95% CI)	*p*
Age (years)				
14–	ref	ref	ref	ref
25–	0.703	0.473	2.02 (0.80–5.10)	0.137
30–	1.675	0.455	5.34 (2.19–13.02)	<0.001
35–	1.832	0.467	6.24 (2.50–15.59)	<0.001
40–	2.422	0.517	11.26 (4.09–31.02)	<0.001
Household type				
Urban	ref	ref	ref	ref
County/township	0.224	0.279	1.25 (0.72–2.16)	0.422
Rural	0.479	0.326	1.62 (0.85–3.06)	0.141
Comorbidity				
Yes	ref	ref	ref	ref
No	−2.188	0.359	0.11 (0.06–0.23)	
Vaccination history				
Unvaccinated	ref	ref	ref	ref
Interval < 5 years	−2.894	1.033	0.06 (0.01–0.42)	0.005
Interval ≥ 5 years	−1.349	0.254	0.26 (0.16–0.43)	
Unknown	−0.141	0.256	0.87 (0.53–1.44)	0.582
History of sharing syringes				
No	ref	ref	ref	ref
Yes	1.945	0.836	7.00 (1.36–36.01)	0.02
Unknown	0.470	0.363	1.60 (0.79–3.26)	0.195
HBV patients/infected persons around				
No	ref	ref	ref	ref
Yes	2.074	0.239	7.96 (4.98–12.72)	
Unknown	0.800	0.253	2.22 (1.36–3.65)	0.002

**Table 7:** Multivariate Logistic Regression Analysis of the Distribution of HBsAb Seropositivity Among Pregnant Women in Nanning City (2024–2025).

Variable	β	S.E.	aOR (95% CI)	*p*
Age (years)				
14–	ref	ref	ref	ref
25–	0.585	0.161	1.79 (1.31–2.46)	
30–	0.412	0.164	1.51 (1.10–2.08)	0.012
35–	0.694	0.179	2.00 (1.41–2.84)	
40–	0.776	0.253	2.17 (1.32–3.57)	0.002
Household type				
Urban	ref	ref	ref	ref
County/township	−0.305	0.140	0.74 (0.56–0.97)	0.030
Rural	−0.503	0.177	0.60 (0.43–0.86)	0.004
Occupation				
Medical staff	ref	ref	ref	ref
Teacher	−0.367	0.327	0.69 (0.37–1.31)	0.261
Enterprise employees	−0.717	0.287	0.49 (0.28–0.86)	0.012
Institutional staff	−0.966	0.350	0.38 (0.19–0.76)	0.006
Commercial service staff	−0.883	0.300	0.41 (0.23–0.74)	0.003
Worker	−1.201	0.410	0.3 (0.13–0.67)	0.003
Self-employed	−0.895	0.257	0.41 (0.25–0.68)	
Housework/unemployed	−0.997	0.306	0.37 (0.20–0.67)	0.001
Farmer	−1.429	0.312	0.24 (0.13–0.44)	
Others	−1.018	0.297	0.36 (0.20–0.65)	0.001
Educational level				
Junior high school and below	ref	ref	ref	ref
High school/vocational school	0.324	0.143	1.38 (1.04–1.83)	0.024
Junior college	0.170	0.143	1.19 (0.90–1.57)	0.235
Bachelor’s degree	0.618	0.150	1.85 (1.38–2.49)	
Master’s/Doctoral degree	1.426	0.510	4.16 (1.53–11.30)	0.005
Monthly household income per capita (CNY)				
<2000	ref	ref	ref	ref
2000–4999	0.261	0.126	1.30 (1.01–1.66)	0.038
5000–9999	0.097	0.160	1.10 (0.81–1.51)	0.544
≥10,000	−0.076	0.250	0.93 (0.57–1.51)	0.762
Comorbidity				
Yes	ref	ref	ref	ref
No	0.805	0.317	2.24 (1.20–4.17)	0.011
Vaccination history				
Unvaccinated	ref	ref	ref	ref
Interval < 5 years	3.148	0.480	23.29 (9.09–59.66)	<0.001
Interval ≥ 5 years	0.621	0.148	1.86 (1.39–2.48)	<0.001
Unknown	−0.001	0.165	1.00 (0.72–1.38)	0.997

**Table 8:** Multivariate Logistic Regression Analysis of the Distribution of HBcAb Seropositivity Among Pregnant Women in Nanning City (2024–2025).

Variable	β	S.E.	aOR (95% CI)	*p*
Age (years)				
14–	ref	ref	ref	ref
25–	0.706	0.220	2.03 (1.32–3.12)	0.001
30–	1.431	0.217	4.18 (2.73–6.41)	<0.001
35–	1.780	0.227	5.93 (3.80–9.25)	<0.001
40–	2.511	0.288	12.32 (7.00–21.68)	<0.001
Educational level				
Junior high school and below	ref	ref	ref	ref
High school/vocational school	−0.065	0.152	0.94 (0.70–1.26)	0.668
Junior college	−0.298	0.155	0.74 (0.55–1.00)	0.054
Bachelor’s degree	−0.403	0.158	0.67 (0.49–0.91)	0.010
Master’s/Doctoral degree	−1.013	0.462	0.36 (0.15–0.90)	0.028
Comorbidity				
Yes	ref	ref	ref	ref
No	−1.224	0.321	0.29 (0.16–0.55)	
Vaccination history				
Unvaccinated	ref	ref	ref	ref
Interval <5 years	−1.448	0.328	0.24 (0.12–0.45)	
Interval ≥5 years	−0.347	0.160	0.71 (0.52–0.97)	0.031
Unknown	−0.093	0.180	0.91 (0.64–1.30)	0.604
Gravidity				
G1	ref	ref	ref	ref
G2	0.023	0.124	1.02 (0.80–1.30)	0.851
G3 or more	0.336	0.137	1.40 (1.07–1.83)	0.014
HBV patients/infected persons around				
No	ref	ref	ref	ref
Yes	0.959	0.153	2.61 (1.93–3.52)	
Unknown	0.164	0.132	1.18 (0.91–1.53)	0.214

**Table 9:** Distribution of HBsAb levels among pregnant women in Nanning under different infection statuses from 2024 to 2025. (mIU/mL).

Variable	N	Uninfected			Previous/Occult Infections		
		*n*	aGMC (95% CI)	aGMR (95% CI)	*n*	aGMC (95% CI)	aGMR (95% CI)
Total	1800	1295	4.63 (3.72–5.75)		505	109.48 (88.22–135.85)	
Age (years)							
14–	245	213	15.48 (3.23–74.16)	ref	26	10.49 (1.47–74.94)	ref
25–	584	452	14.82 (4.60–47.78)	0.96 (0.37–2.45)	109	78.15 (23.85–256.06)	7.45 (2.07–26.84)
30–	614	387	2.48 (0.77–7.98)	0.16 (0.04–0.7)	177	111.06 (46.32–266.28)	10.58 (1.85–60.47)
35–	386	205	1.50 (0.34–6.6)	0.10 (0.01–0.77)	141	118.28 (43.47–321.85)	11.27 (1.10–115.12)
40–	106	38	0.94 (0.10–8.69)	0.06 (0–1.08)	52	96.74 (19.98–468.27)	9.22 (0.49–174.45)
Household type							
Urban	854	580	10.67 (3.76–30.32)	ref	225	115.24 (48.45–274.09)	ref
County/township	776	523	4.13 (1.46–11.68)	0.39 (0.21–0.71)	201	100.44 (42.96–234.85)	0.87 (0.50–1.51)
Rural	305	192	2.65 (0.87–8.05)	0.25 (0.11–0.54)	79	72.78 (28.69–184.65)	0.63 (0.30–1.34)
Ethnicity							
Han	1002	668	6.34 (2.08–19.32)	ref	269	88.42 (38.81–201.45)	ref
Zhuang	872	580	4.71 (1.54–14.35)	0.74 (0.47–1.17)	223	113.93 (49.89–260.18)	1.29 (0.81–2.04)
Others	61	47	9.33 (2.02–43.04)	1.47 (0.48–4.54)	13	16.41 (3.41–78.85)	0.19 (0.05–0.74)
Occupation							
Medical staff	128	88	33.24 (9.34–118.29)	ref	34	210.15 (69.55–635)	ref
Teacher	111	77	13.59 (3.72–49.70)	0.41 (0.13–1.30)	30	81.92 (24.75–271.13)	0.39 (0.11–1.35)
Enterprise employees	205	147	7.11 (2.14–23.58)	0.21 (0.08–0.58)	46	100.10 (34.08–294.03)	0.48 (0.16–1.44)
Institutional staff	77	51	5.20 (1.23–22.00)	0.16 (0.04–0.57)	19	60.29 (15.18–239.45)	0.29 (0.07–1.16)
Commercial service staff	151	91	3.64 (1.00–13.20)	0.11 (0.04–0.33)	49	118.93 (41.98–336.99)	0.57 (0.19–1.70)
Worker	42	23	2.17 (0.34–13.65)	0.07 (0.01–0.36)	14	50.29 (10.68–236.78)	0.24 (0.05–1.14)
Self-employed	809	541	4.41 (1.51–12.93)	0.13 (0.06–0.32)	210	84.44 (36.36–196.13)	0.40(0.16–1.02)
Housework/unemployed	126	89	5.56 (1.51–20.40)	0.17 (0.05–0.52)	30	122.41 (39.57–378.65)	0.58 (0.17–1.98)
Farmer	130	79	1.02 (0.28–3.72)	0.03 (0.01–0.10)	38	44.84 (14.40–139.66)	0.21 (0.07–0.70)
Others	156	109	4.31 (1.24–14.97)	0.13 (0.04–0.38)	35	49.51 (15.68–156.35)	0.24 (0.07–0.78)
Educational level							
Junior high school and below	535	321	1.83 (0.63–5.33)	ref	167	82.39 (35.49–191.23)	ref
High school/vocational school	393	256	4.01 (1.33–12.06)	2.19 (1.18–4.07)	110	149.21 (60.11–370.36)	1.81 (0.98–3.36)
Junior college	453	322	6.10 (2.06–18.01)	3.33 (1.83–6.05)	98	70.17 (28.01–175.74)	0.85 (0.45–1.63)
Bachelor’s degree	521	370	13.44 (4.67–38.62)	7.33 (4.03–13.34)	123	131.68 (53.64–323.28)	1.60 (0.86–2.97)
Master’s/Doctoral degree	33	26	20.52 (3.72–113.08)	11.2 (2.44–51.47)	7	117.93 (15.74–883.54)	1.43 (0.22–9.44)
Monthly household income per capita (CNY)							
<2000	400	258	3.07 (0.98–9.62)	ref	104	64.76 (26.86–156.11)	ref
2000–4999	1093	734	6.25 (2.12–18.49)	2.04 (1.18–3.53)	295	128.8 (57.2–290.01)	1.99 (1.14–3.47)
5000–9999	351	243	7.21 (2.31–22.54)	2.35 (1.18–4.68)	83	72.15 (27.6–188.62)	1.11 (0.54–2.30)
≥10,000	91	60	5.11 (1.21–21.66)	1.67 (0.56–4.94)	23	46.38 (12.84–167.51)	0.72 (0.23–2.21)
Comorbidity							
Yes	47	17	5.11 (0.76–34.25)	ref	12	103.23 (24.12–441.73)	ref
No	1888	1278	5.93 (2.62–13.46)	1.16 (0.19–7.13)	493	93.22 (60.82–142.89)	0.90(0.22–3.76)
Vaccination history							
Unvaccinated	237	148	0.94 (0.27–3.28)	ref	57	129.86 (50.53–333.7)	ref
Interval < 5 years	106	92	177.3 (48.06–654.11)	188.15 (69.71–507.86)	13	57.46 (12.48–264.58)	0.44 (0.10–1.99)
Interval ≥ 5 years	1164	778	4.15 (1.37–12.57)	4.41 (2.25–8.62)	336	118.86 (56.75–248.95)	0.92 (0.46–1.84)
Unknown	428	277	1.32 (0.42–4.21)	1.41 (0.66–2.99)	99	104.41 (45.36–240.34)	0.80 (0.36–1.81)
Gravidity							
G1	833	604	6.96 (2.31–20.99)	ref	172	96.45 (40.87–227.64)	ref
G2	647	433	4.79 (1.58–14.55)	0.69 (0.43–1.11)	170	78.16 (33.42–182.78)	0.81 (0.47–1.38)
G3 or more	455	258	3.53 (1.09–11.44)	0.51 (0.28–0.91)	163	127.79 (54.23–301.13)	1.32 (0.76–2.32)
Surgical history							
No	1282	868	5.90 (1.87–18.63)	ref	329	94.72 (40.90–219.37)	ref
Yes	584	377	4.37 (1.36–14.04)	0.74 (0.47–1.18)	161	102.03 (44.61–233.35)	1.08 (0.67–1.74)
Unknown	69	50	26.12 (5.63–121.04)	4.43 (1.49–13.2)	15	80.55 (18.2–356.54)	0.85 (0.23–3.11)
Blood transfusion history							
No	1711	1153	5.45 (1.79–16.64)	ref	441	99.69 (44.44–223.67)	ref
Yes	144	91	5.36 (1.42–20.19)	0.98 (0.44–2.21)	42	70.12 (24.39–201.60)	0.70 (0.32–1.56)
Unknown	80	51	8.91 (1.95–40.69)	1.63 (0.56–4.8)	22	142.51 (39.85–509.72)	1.43 (0.48–4.22)
Acupuncture history							
No	1444	974	5.12 (1.69–15.56)	ref	376	104.3 (46.45–234.18)	ref
Yes	417	272	6.91 (2.16–22.11)	1.35 (0.81–2.26)	111	70.08 (28.52–172.24)	0.67 (0.40–1.14)
Unknown	74	49	4.00 (0.87–18.42)	0.78 (0.26–2.36)	18	147.3 (37.36–580.83)	1.41 (0.42–4.71)
Syringe sharing history							
No	1838	1240	5.34 (1.74–16.38)	ref	478	99.15 (44.6–220.4)	ref
Yes	9	4	0.50 (0.01–24.16)	0.09 (0–3.87) *	2	940.92 (27.28–32,451.22)	9.49 (0.30–301.87) *
Unknown	88	51	9.03 (2.02–40.41)	1.69 (0.58–4.96)	25	73.14 (20.97–255.12)	0.74 (0.26–2.06)
Shared tableware & daily supplies							
Frequently	46	33	3.55 (0.67–18.88)	ref	10	108.25 (20.09–583.32)	ref
Occasionally	399	276	5.30 (1.64–17.15)	1.50 (0.38–5.89)	94	151.46 (59.55–385.23)	1.4 (0.28–7.09)
Never	1490	986	5.70 (1.86–17.45)	1.61 (0.43–6.00)	401	90.64 (40.64–202.19)	0.84 (0.18–4.00)
HBV patients/infected persons around							
No	1274	898	4.77 (1.57–14.45)	ref	329	84.31 (37.14–191.41)	ref
Yes	251	117	11.97 (3.40–42.09)	2.51 (1.20–5.26)	79	117.45 (46.6–296.01)	1.39 (0.76–2.56)
Unknown	410	280	4.98 (1.53–16.16)	1.04 (0.63–1.74)	97	121.93 (49.21–302.14)	1.45 (0.82–2.55)

Note: * Statistical estimation and geometric calculation are unstable for sample sizes of n < 5.

## Data Availability

The raw data supporting the conclusions of this article will be made available by the corresponding author upon reasonable request. The authors declare that no generative AI was used in the creation of this manuscript.

## Supplementary Materials

The following supporting information can be downloaded at: https://www.mdpi.com/article/10.3390/vaccines14090765/s1, Table S1: Distribution of HBV Serological Markers by Clinical Exposure Among Pregnant Women in Nanning City, 2024–2025; Table S2: Variable Assignment Table for the Multivariate Logistic Regression Model.
