# Supplementary material for: Seroepidemiological Characteristics and Influencing Factors of Hepatitis B Virus Infection Among Pregnant Women in Nanning City, China (2024–2025): A Cross-Sectional Study
Source: Vaccines (Basel). 2026 Sep 1;14(9):765. doi: 10.3390/vaccines14090765 (PMC13611315; doi:10.3390/vaccines14090765)
Supplement: Supplementary file 1 [file vaccines-14-00765-s001.zip › vaccines-4499147-supplementary.pdf]

Table S1. Distribution of HBV Serological Markers by Clinical Exposure Among Pregnant Women in Nanning City , 2024–2025.

Var	N	HBsAg			HBsAb			HBeAg			HBeAb			HBcAb		
		n	rate%(95%CI)	*p*	*n*	rate%(95%CI)	*p*	*n*	rate%(95%CI)	*p*	*n*	rate%(95%CI)	*p*	*n*	rate%(95%CI)	*p*
Total	1935	135	6.98(5.92–8.20）		1151	59.48(57.28–61.65)		29	1.50(1.05–2.14)		300	15.50(13.96–17.18)		639	33.02(30.96–35.15)	
Surgical history																
No	1282	85	6.63(5.40 – 8.12)	ref	764	59.59(56.89 – 62.24)	ref	15	1.17(0.71 – 1.92)	ref	201	15.68(13.8 – 17.77)	ref	413	32.22(29.72 – 34.82)	ref
Yes	584	46	7.88(5.97 – 10.33)	0.329	340	58.22(54.20 – 62.13)	0.575	14	2.4(1.44 – 3.98)	0.052	91	15.58(12.89 – 18.73)	0.958	207	35.45(31.7 – 39.38)	0.170
Unknown	69	4	5.8(2.59 – 13.67)	0.786	47	68.12(56.98 – 77.34)	0.161	0	0(0.14 – 5.13)*	0.986	8	11.59(6.40 – 20.84)	0.363	19	27.54(18.94 – 38.5)	0.418
Blood transfusion history																
No	1711	117	6.84(5.74 – 8.13)	ref	1018	59.5(57.16 – 61.79)	ref	25	1.46(0.99 – 2.15)	ref	266	15.55(13.91 – 17.34)	ref	557	32.55(30.38 – 34.81)	ref
Yes	144	11	7.64(4.43 – 13.05)	0.716	86	59.72(51.77 – 67.17)	0.958	3	2.08(0.78 – 5.88)	0.559	19	13.19(8.76 – 19.55)	0.453	53	36.81(29.57 – 44.73)	0.298
Unknown	80	7	8.75(4.59 – 16.69)	0.511	47	58.75(48.29 – 68.41)	0.894	1	1.25(0.37 – 6.6)	0.877	15	18.75(12.09 – 28.27)	0.442	29	36.25(27.04 – 46.72)	0.491
Acupuncture history																
No	1444	94	6.51(5.35 – 7.90)	ref	851	58.93(56.38 – 61.44)	ref	19	1.32(0.85 – 2.04)	ref	224	15.51(13.74 – 17.47)	ref	469	32.48(30.12 – 34.93)	ref
Yes	417	34	8.15(5.92 – 11.15)	0.244	261	62.59(57.89 – 67.06)	0.180	8	1.92(0.99 – 3.73)	0.367	64	15.35(12.24 – 19.09)	0.935	145	34.77(30.40 – 39.42)	0.380
Unknown	74	7	9.46(4.99 – 17.93)	0.324	39	52.70(42.02 – 63.11)	0.290	2	2.7(0.96 – 9.12)	0.330	12	16.22(9.94 – 25.83)	0.871	25	33.78(24.57 – 44.6)	0.815
Shared tableware & daily supplies																
Frequently	46	3	6.52(2.83 – 16.91)	ref	26	56.52(43.31 – 68.73)	ref	2	4.35(1.71 – 14.02)	ref	8	17.39(9.92 – 29.89)	ref	13	28.26(18.3 – 41.58)	ref
Occasionally	399	29	7.27(5.13 – 10.22)	0.853	236	59.15(54.31 – 63.82)	0.732	9	2.26(1.21 – 4.22)	0.395	56	14.04(11.00 – 17.75)	0.540	123	30.83(26.54 – 35.48)	0.721
Never	1490	103	6.91(5.74 – 8.31)	0.918	889	59.66(57.16 – 62.12)	0.669	18	1.21(0.77 – 1.90)	0.084	236	15.84(14.08 – 17.78)	0.777	503	33.76(31.41 – 36.19)	0.438

Note: * Statistical estimates with 95% CIs are unstable for sample sizes of n < 5.

Table S2. Variable Assignment Table for the Multivariate Logistic Regression Model.

**variable**	**Value Explanation**
HBsAg	Negative=0, Positive=1
HBsAb	Negative=0, Positive=1
HBcAb	Negative=0, Positive=1
Age (years)	14–years = 0; 25–years = 1; 30–years = 2; 35–years = 3; 40–years = 4
Residential area	Urban = 0, County/township = 1, Rural = 2

Occupation	Medical staff = 0; Teachers = 1; Enterprise employees = 2; Institutional staff = 3; Commercial service staff = 4; Workers = 5; Self-employed = 6; Housework/unemployed = 7; Farmers = 8; Others = 9
Educational	Junior high school or below = 0; High school/vocational school= 1; Junior college = 2; Bachelor's degree = 3; Master's/Doctoral degree = 4
Monthly household per capita income (CNY)	<2000= 0; 2000-4999 = 1; 5000-9999 = 2; ≥10000 = 3
Comorbidity	Yes = 0, No = 1
Vaccination history	Unvaccinated = 0; Immunization interval <5 years = 1; Immunization interval ≥ 5 years = 2; Unknown = 3
Number of pregnancies	G1= 0; G2= 1; G3 or more = 2
History of sharing syringes	Yes = 0, No = 1, Unknown = 2
There are hepatitis B patients around me.	Yes = 0, No = 1, Unknown = 2
